# Pt_12_H_24_^−^: A Cuboctahedral Platinum Hydride Cluster Cage

**DOI:** 10.1002/cphc.202400649

**Published:** 2024-10-28

**Authors:** Siddhi Gojare, Dennis Bumüller, Stephan Kohaut, Manuel Kraft, Ulrich Heiz, Manfred M. Kappes, Karin Fink, Detlef Schooss

**Affiliations:** ^1^ Institute of Nanotechnology Karlsruhe Institute of Technology (KIT) Kaiserstraße 12 76131 Karlsruhe Germany; ^2^ Chair of Physical Chemistry and Catalysis Research Center Department of Chemistry School of Natural Sciences Technical University of Munich 85748 Garching Germany; ^3^ Institute of Physical Chemistry Karlsruhe Institute of Technology (KIT) Kaiserstraße 12 76131 Karlsruhe Germany

**Keywords:** platinum, hydrides, cluster compounds, electron diffraction, density functional theory

## Abstract

The platinum hydride cluster Pt_12_H_24_^−^ is studied in gas phase by a combination of trapped ion electron diffraction and density functional theory computations. We find a cuboctahedral platinum cage with bridge bound hydrogen atoms. This unusual structure is stabilized by Pt‐H‐Pt multicenter bonds and shows characteristics of spherical aromaticity.

## Graphical abstract

A combination of trapped ion electron diffraction and density functional theory indicate the hydrogen induced structure change of Pt^−^_12_ from a truncated bipyramid to a cuboctahedral cage structure. The Pt‐cage structure is stabilized by hydrogen 2e‐3c bridge bonds and spherical aromaticity.

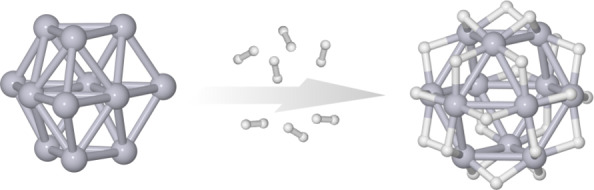

## Introduction

Hydrogen is ubiquitous in many noble metal catalyzed reactions and either directly involved as an educt/reaction product or indirectly as a hydride intermediate. The interaction of hydrogen with subnanometer Pt particles has attracted attention recently.[^1^, ^2^, ^3^] On extended Pt surfaces, the hydride formation is well studied and largely understood.[^4^, ^5^, ^6^] Hydrogen induced structural modifications have been observed experimentally for supported Pt nanoparticles, where an increase of the mean Pt‐Pt distance and coordination number was found.[^1^, ^2^, ^7^] Hydride formation on supported Pt clusters is complicated by the complex interplay of cluster‐adsorbate, cluster‐support and adsorbate‐support interactions. Insights on the processes involved were obtained by density functional theory (DFT) based investigations for small Pt‐clusters,[^8^, ^9^, ^10^] which suggest major reconstruction under formation of Pt‐hydride clusters, isolated in gas phase and on supports. However, direct or indirect experimental verification of this structural transformation is hardly available.

**Figure 1:**
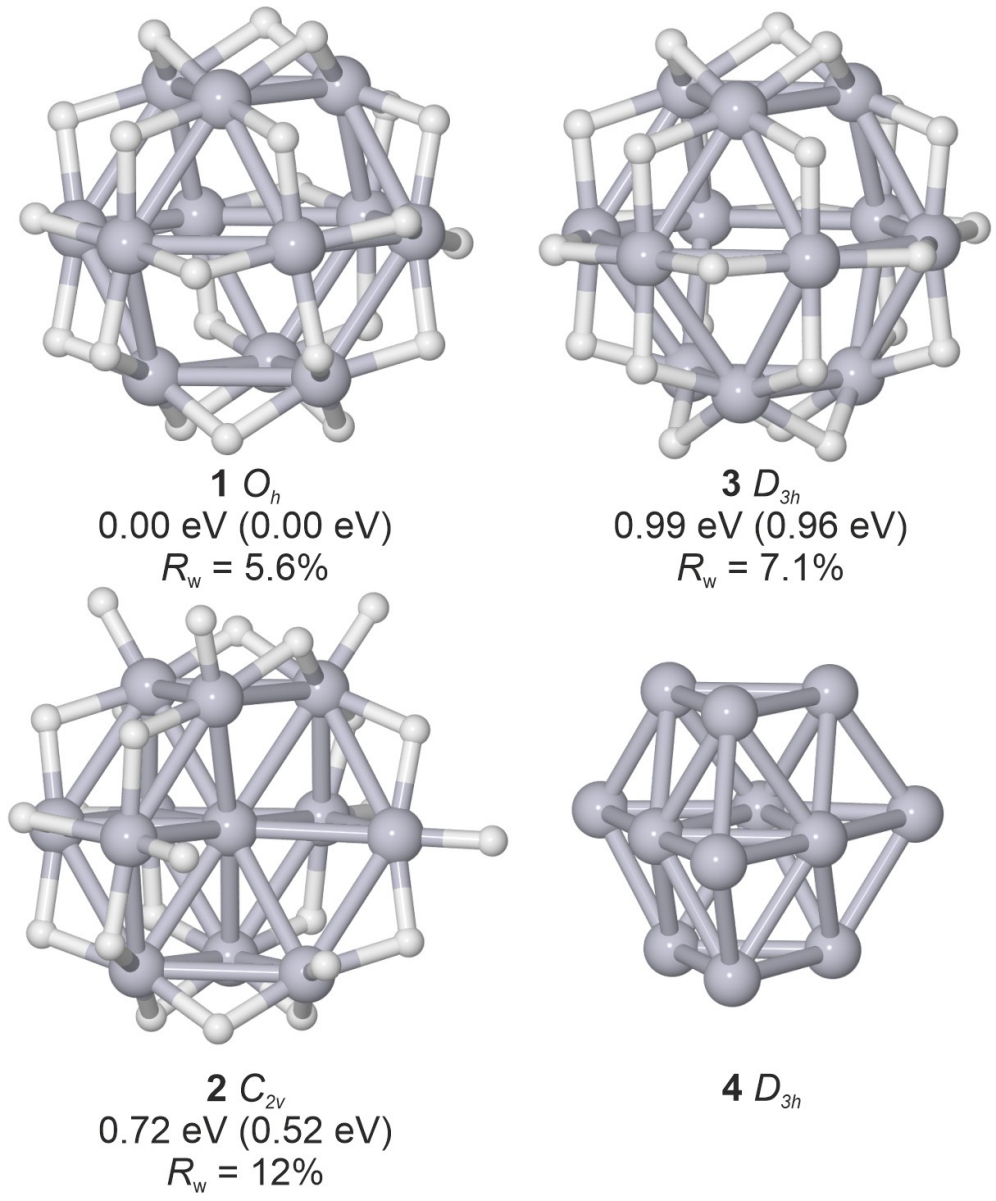
Computed structures of Pt_12_D_24_^−^ isomers **1**–**3** with the approximate point group symmetry for the Pt‐core, relative energy for TPSS/def2‐TZVP (2C‐TPSS/dhf‐TZVP‐2c), and experimental profile factor. Structure **4** shows the bare cluster ${\mathrm{Pt}}_{12}^{-}$ .

A possible route to disentangle intrinsic from surface and collective effects, is studying the properties of well‐defined, isolated Pt clusters in gas phase. In a recent study, we have determined the structural evolution of free ${\mathrm{Pt}}_{n}^{-}$ *n*=6–13 clusters by a combination of trapped ion electron diffraction and DFT computations.^[11]^ For ${\mathrm{Pt}}_{12}^{-}$ a doubly truncated trigonal bipyramid (Figure 1, **4**) was found based on the diffraction data. In this work, we investigate the effect of the hydrogen exposition of ${\mathrm{Pt}}_{12}^{-}$ on its structure in gas phase using the same methodology. Instead of 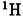 we use deuterium throughout this study for experimental reasons.

## Methods

Structural assignments using TIED is based on the comparison of experimental and simulated molecular scattering functions from candidate structures. The TIED experiment has been described in detail elsewhere.[^12^, ^13^] Platinum hydride cluster anions were generated by a magnetron sputter source^[14]^ with deuterium (0.3 vol %) in the sputter gas at a total pressure of 500 Pa and a temperature of ≈120 K. The equilibrium stoichiometry under these conditions was obtained from a TOF‐mass spectrum (Figure S1).^[15]^ Pt_12_D_24±1_^−^ cluster anions were isolated by a quadrupole mass filter, captured in a cooled (95±5 K) quadrupole ion trap and thermalized by collisions with about 1 Pa He for several seconds. A 40 keV electron beam crossed the ion cloud consisting of ca. 10^5^ to 10^6^ clusters. Electrons scattered during exposure periods of 20–30 s were detected on a phosphor screen assembly and integrated on an external charge‐coupled device (CCD) camera. The scattering function is largely dominated by the Pt core and variations of the deuterium coverage can be only observed indirectly by changes induced in the platinum core structure. To quantify the agreement between the experimental molecular scattering function and the simulated ones from candidate structures, we use the weighted profile factor *R_w_*. For its definition and further details of the methodology, please see the SI.

Structural models for TIED fitting were obtained in a multistep procedure. Semiempirical potential energy functions[^16^, ^17^, ^18^] were used within a modified genetic algorithm to generate well‐fitting core structures.^[19]^ The geometries of the best fitting / the lowest energy isomers were re‐optimized using DFT computations within the TURBOMOLE package (version 7.7).[^20^, ^21^]

Based on a comparison of simulated and experimental scattering data, three different Pt core structures were selected for the further optimization of the deuterium configuration using the TPSS functional together with the def2‐TZVP basis set. A previous study on ${\mathrm{Pt}}_{n}^{-}$ clusters showed that spin‐orbit contributions (SOC) can have a significant influence on the relative energies and the geometry of the isomers for bare clusters.^[11]^ We tested for the effect of SOC using a relativistic two‐component method^[22]^ with the TPSS functional in combination with the dhf‐TZVP‐2c basis set. For further details of the computational methods, please see the SI. Relative energies and adsorption energies include zero point vibrational energy corrections unless otherwise stated.

## Results and Discussion

Figure 1 shows the lowest energy isomers for each of the three Pt‐core structures. Structure **1** is a cuboctahedral cage structure. The 24 deuterium atoms are bridging the 24 Pt‐Pt bonds, arranged in a way that the full symmetry (*O*_h_) is unchanged. D configurations with on‐top bonds are also stable but have significantly higher energy (>1.1 eV, see Table S1).

The relative energy of isomer **2** is 0.72 eV. Its Pt‐core structure is an incomplete cuboctahedron where an atom is missing in the 12‐atom cuboctahedral shell, leading to a ≈*C*_2V_ symmetry for the Pt‐core. With regard to the deuterium configuration, several close‐lying isomers with different distributions of bridge and on‐top bound deuterium have been found (see Table S2). The lowest energy isomer within this structure family is shown in Figure 1 and contains 18 bridge and six on‐top bound deuterium atoms.

Finally, structure **3** is another cage isomer, where the bottom triangle of isomer **1** is rotated by 60°, forming an anti‐cuboctahedral cage with approximate *D*_3h_ symmetry. It is 0.99 eV higher in energy and similar to **1** all deuterium atoms are in bridge positions decorating the Pt‐Pt bonds. It is only stable for an all bridged configuration, and introducing on‐top bound D leads to a reconstruction of the **3** core.

The Pt‐cores of isomers **1** and **2** belong to the same structural motif, they vary only by occupying different positions of the 13 atom cuboctahedron. In isomer **3** only the layer order is modified from fcc to hcp stacking. Thus, the molecular scattering functions of the three isomers are expected to be similar.

**Figure 2:**
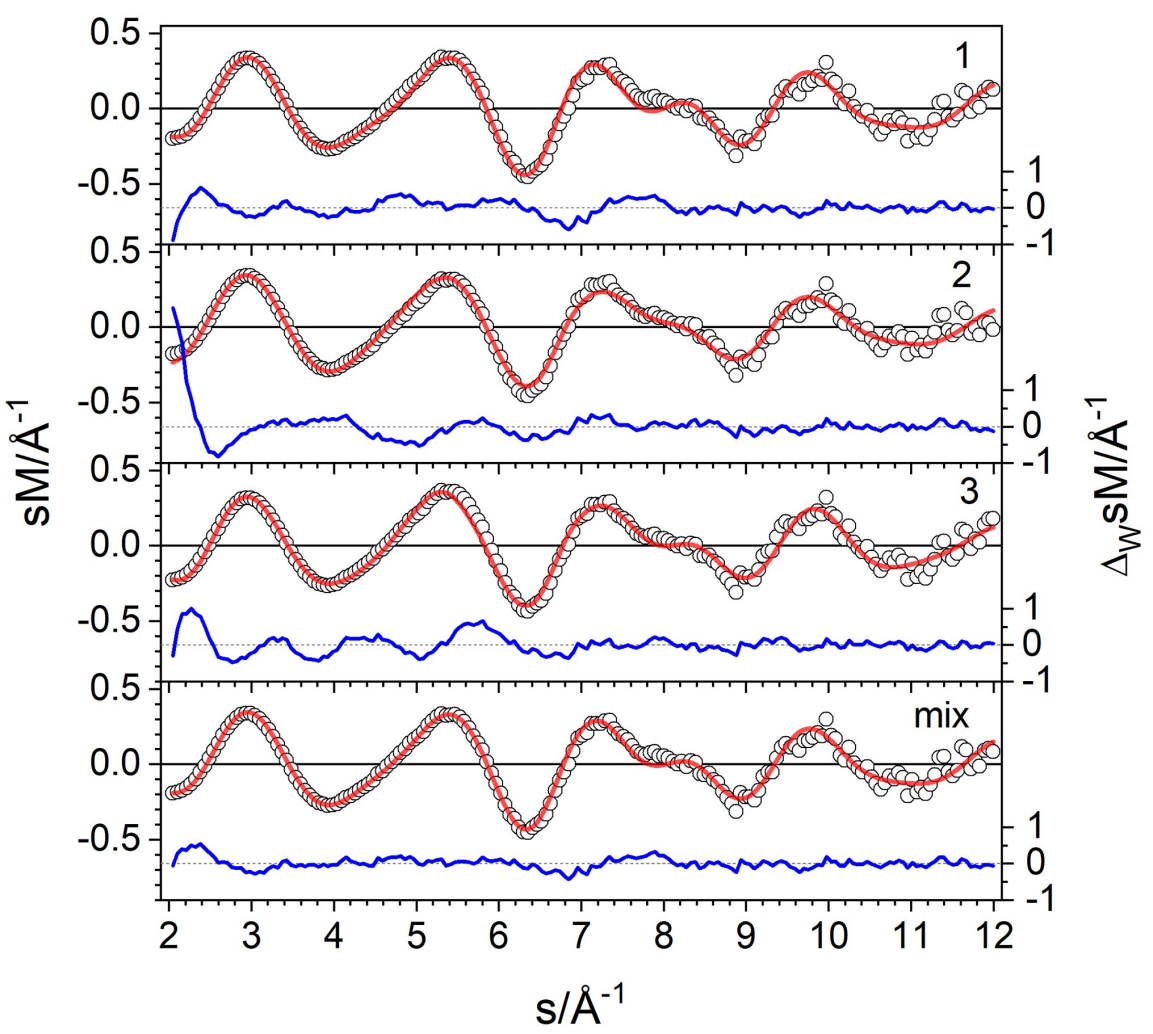
Comparison of experimental (circles) and simulated (red line) modified molecular scattering intensities *sM* of Pt_12_D_24±1_^−^ for the isomers **1**–**3** and a mixture of 80 % isomer **1** and 20 % isomer **2**. The blue line in each panel shows the weighted residuals.

Nevertheless, we find a clear preference for the isomer **1** with a profile factor of $R_{w}$ =5.6 % in the TIED experiment (Figure 2). However, isomers with different D configurations ‐ mixtures of bridge and on‐top bound D ‐ show comparable or even lower profile factors (Table S1) but can be ruled out based on relative energy. The *R_w_* values for isomers with a different Pt‐core structure **2** and **3** are significantly higher, 12 % and 7.1 %, respectively. The large difference in the *R_w_* value of **1** and **2** is caused by specific deviation primarily in the small *s* range due to the cage nature of **1** which are amplified by the larger statistical weighting in this region (blue line in Figure 2). Based on the profile factor, we assign isomer **1** as the dominant contributor of the ensemble probed. However, we cannot rule out a small contribution from isomer **2**, as in a mixture fit with ≈20 % isomer **2**, the profile factor is reduced to 4.3 % (see Figure 2). Isomers with different deuterium distributions on the **2** core lead to similar results in the mixture fit procedure.

Before considering the possible reasons for the high stability of the cage structure **1** in detail, the influence of charge, spin‐orbit coupling and the finite temperature of the experiment will be examined.

Reoptimizations for the neutral ${\mathrm{Pt}}_{12}D_{24}$ , cationic Pt_12_D_24_^+^, and dianionic isomers show no significant structural changes. However, the charge state has an influence on the energetic order: while for the neutral clusters and dianionic clusters **1** is still the most stable structure, for the cationic system **2** is the lowest energy structure within these isomers (Table S3).

Inclusion of SOC for the anionic clusters (Pt_12_D_24_^+^) decreases the relative energy differences, but the same energetic ordering is maintained. The Pt‐Pt bond length of isomer **1** decreased from 2.716 Å to 2.702 Å after the inclusion of SOC, in agreement with the results of former studies.[^8^, ^23^, ^24^] Neither consideration of the finite experimental temperature nor the inclusion of dispersion changes the relative energy differences significantly (Table S4).

For the minor component, isomer **2**, different deuterium configurations similar in energy were found, suggesting a dynamical structure at the experimental temperature of 120 K. The mobility of deuterium atoms on the cluster surface is largely determined by activation barriers connected to their binding mode. We have estimated the activation barriers for different D configurations by nudge elastic band calculations: typical activation energies are in the range of 0.5 eV. A sequential rearrangement of deuterium atoms on the cluster surface of isomer **2** can, in addition, induce the transformation to isomer **1**, coupling the deuterium mobility with the core isomerization. A possible route is shown in Figure S3. Six consecutive steps were necessary for the conversion from isomer **2** to **1** with an overall activation barrier of 1.4 eV. The latter did not change after the inclusion of the SOC effect. The results indicate that at the experimental condition, an isomerization within the structure family of **2** is possible, however a dynamic interconversion of isomers **1** and **2** is unlikely.

The cause of the improved stability of the cuboctahedral cage can be separated energetically to first order into two parts: (i) the relative contribution of the core structures, and (ii) the contribution of the respective deuterium shells.

To evaluate the first part, we removed all deuterium atoms from the cluster and recalculated the (single‐point) energy of the remaining ${\mathrm{Pt}}_{12}^{-}$ cores. The core of **2** is lowest in energy, as expected, since **2** has the highest mean coordination number (CN) of 5.16 while the CN of the cage structures **1** and **3** is only 4.00 leading to a destabilization of 0.43 and 1.16 eV, respectively. Please note, that these ${\mathrm{Pt}}_{12}^{-}$ core structures do not represent stable configurations for the bare cluster.

To characterize the deuterium shell, we use the mean as well as the single deuterium adsorption energy as defined in Eqs. (1) and (2), respectively.
(1)

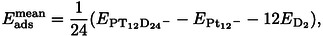



(2)

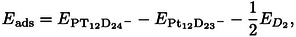




with $E_{{\mathrm{Pt}}_{12}^{-}}$ the energy of isomer **4**.

The mean adsorption energy per deuterium atom (Table 1) is highest for isomer **1** followed by **2** and **3** reflecting their relative stability. Note, that the mean adsorption energies are in the same energy range as those proposed for Pt surfaces, but depending on the surface structure, hollow sites positions are the most stable there.^[6]^

**Table 1:** Deuterium adsorption energies in eV.

isomer	$E_{\mathrm{ads}}^{\mathrm{mean}}$	$E_{\mathrm{ads}}$	
		bridge	on‐top
**1**	−0.50	−1.20	–
**2**	−0.48	−1.22 ‐ −0.33	−0.55 ‐ −0.23
**3**	−0.46	*	–

* Core reconstruction upon removal of one D atom.

The maximum deuterium adsorption energy is around 1.2 eV for the bridged type, while the on‐top deuterium atoms in isomer **2** are significantly less stable (0.55 eV). The adsorption energies of isomer **2** are site‐dependent, for an overview, see Table S5. Adsorption of the deuterium atom in the hollow site at the central Pt atom of isomer **2** is also possible, but results in an even lower adsorption energy.

**Figure 3:**
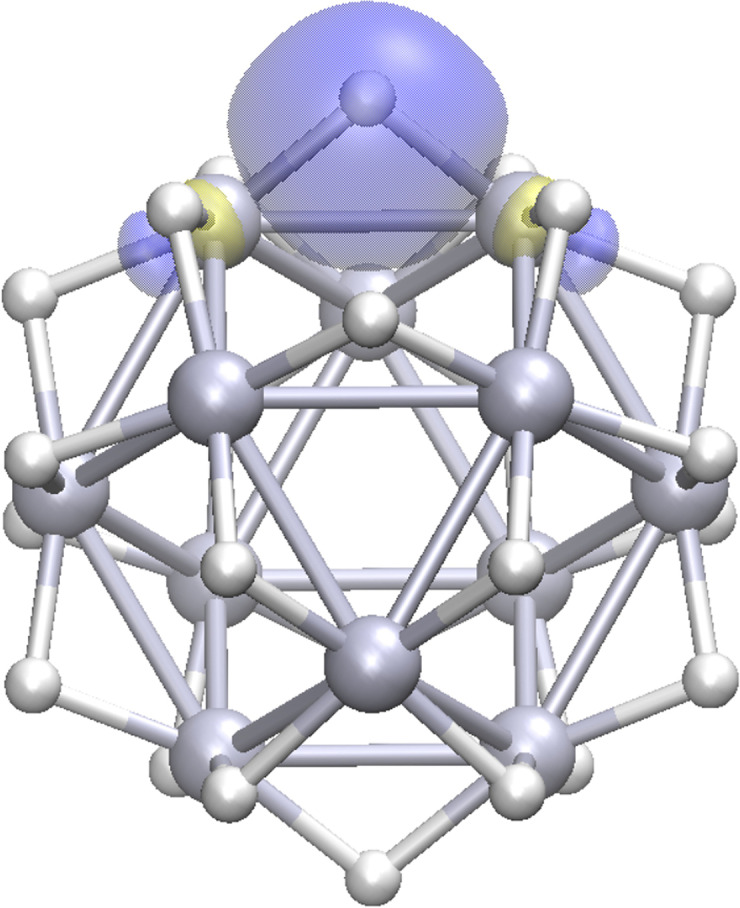
Three center ‐ two electron bonds between Pt‐D‐Pt obtained by Boys localisation for isomer **1**.

To understand the differences in adsorption energy of the two binding modes, we further analyzed the electronic interaction of deuterium with the Pt cluster. For the bare ${\mathrm{Pt}}_{12}^{-}$ , the negative charge is mainly distributed on the apex atoms (Table S6). For isomer **1** the total charge on the core is reversed to ≈+1.5 e. Conversely, the bridge‐bound deuterium atoms show a significant hydridic character of −0.1 e per deuterium compared to −0.05 e for on‐top D atoms in isomer **2** (see Tables S7 and S8). An explanation for the increased interaction of the bridge bound D with the Pt core is its multi‐center‐bond character. Both natural population analysis and the Wiberg index analysis (Wiberg index of 0.82 for each Pt‐D bond) indicate 3c‐2e bonds for the Pt‐D‐Pt bridges stabilizing the cage structures in isomer **1** (and **3**). The visualization of a typical Boys localized Pt‐D‐Pt orbital is shown in Figure 3. The on‐top D in isomer **2**, however, are best described by 2c‐2e bonds (Figure S4).

**Figure 4:**
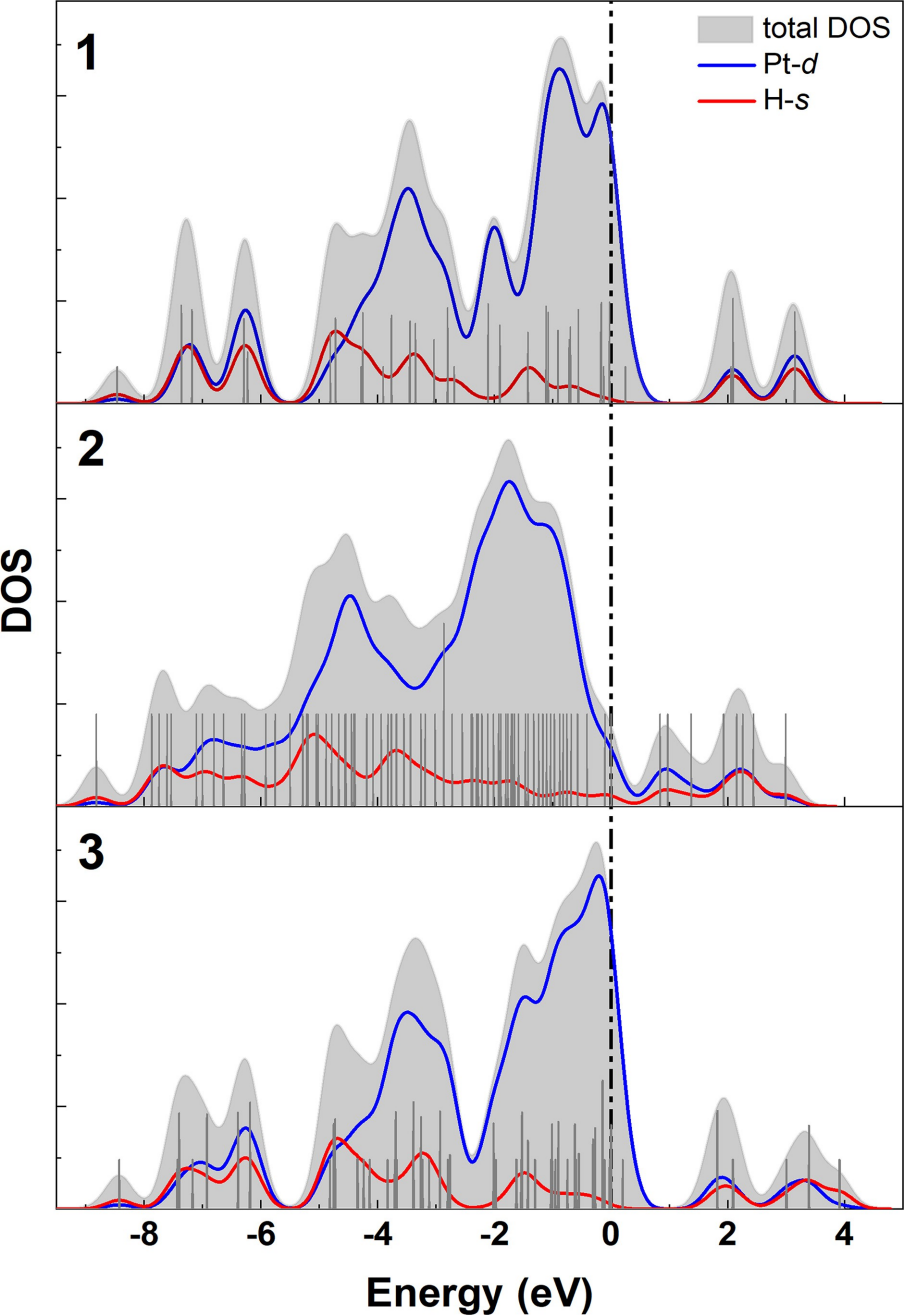
Density of States plot (DOS) of isomers **1**, **2** and **3**. The total density is shown in gray, the contributions from the Pt‐5*d* and H‐1*s* atomic orbitals are marked blue and red, respectively. The Fermi level is indicated as a dashed vertical line.

Further insights can be gained from the comparison of the density of states shown in Figure 4. In all three isomers, the Pt *5d* orbitals dominate the valence band close to the Fermi level. In contrast to **2**, the D‐*1s* contribution is negligible near the Fermi level for the cage structures.

The *d*‐band center considering the Pt‐5*d* orbital contributions of all Pt‐surface atoms amounts up to −2.04 eV for the structure **1** and to −2.26 eV for **3**. Following the *d*‐band‐center rule of Hammer and Nørskov,^[25]^ that the interaction with deuterium is stronger the closer the metal *d* states are located to the Fermi level, we conclude that the higher mean hydrogen binding energy of isomer **1** originates from its more favorable electronic structure, even though like in isomer **1**, all deuterium atoms are bridge bound in isomer **3**.

Pt_12_D_24_^−^ is an open shell system. However, the cage structures show a remarkably large band gap if the broadened DOS is considered. The band gaps for **1**–**3** are 1.99, 0.84 and 1.71 eV, respectively. Because of the odd number of electrons, a gap state can occur in the minority spin channel in unrestricted DFT calculations. Such empty orbitals are observed at 0.25, 0.56 and 0.20 eV for **1**–**3**. In the corresponding close shell computation of Pt_12_D_24_^2−^ the band gaps change only slightly to 1.73, 0.40 and 1.56 eV for isomers **1**–**3**, respectively (see SI Figure S5).

Large band gaps indicate electronically closed shell configurations, most obviously for example in the spherical jellium free electron gas model for simple clusters.^[26]^ A similar model exists for a free electron gas on a spherical shell,^[27]^ in chemistry better known as the Hirsch rule^[28]^ for spherical (π) aromaticity.

The Pt atoms in **1** are distributed over a spherical shell. Ignoring *sd* hybridization and assuming that per D atom a full electron is transferred, the total number of valence Pt‐5*d* electrons for Pt_12_H_24_^2−^ is 98 satisfying the shell closure condition $n=2{\left(N+1\right)}^{2}$ for N=6. Accordingly, the monoanion Pt_12_H_24_^−^ with 97 electrons represents a near electronic shell closure. For both systems, we scrutinized for spherical aromaticity by computing the Nucleus Independent Chemical Shift (NICS) value. It has been calculated along one of the cluster's *C_3_* axes at 1 Å away above the deuterium plane (Figure S6) to avoid the contamination of σ‐electrons.^[29]^ A NICS value of −5.67 ppm has been calculated for isomer **1** and −6.55 ppm for **1** ^2−^, indicating a sizable aromatic character in both cases. Additionally, the structural analysis of the isomer **1** revealed, that all Pt‐Pt distances (2.71 Å) are equal. Such bond equalization indicates all‐metal aromaticity,^[30]^ which stabilizes the cuboctahedral cage. The latter stabilization is absent in isomer **3** which has a distribution of Pt‐Pt distances (see Figure S7) which rationalizes the energy difference between isomers **1** and **3**. Finally, in isomer **2** some of the bridge bound deuterium atoms are replaced by on‐top ones, leading to an increase of the mean bond length (2.77 Å).

## Conclusions

In summary, we have used trapped ion electron diffraction along with density functional theory calculations to investigate the underlying reasons for the stability of the cuboctahedral cage structure of Pt_12_H_24_^−^. We have shown, that the cuboctahedral cage is the most probable isomer for Pt_12_H_24_^−^: isomer **1** has the lowest profile factor and is the lowest energy isomer, independent of the computational method used. However, the stability of **1** is counterintuitive on first sight, because bare metal clusters usually prefer structures with high CN. Instead, the number of Pt‐Pt bonds in the cage **1** is significantly lower than in isomer **2**. Obviously, in these deuteride structures, the number and type of Pt‐D contacts is equally or even more important. The results of the structural analysis of isomer **1** shows that bridged bound deuterium atoms act as staples stabilizing the cage structure due to the strong interactions with the neighboring Pt atoms by multi center bonds, resulting in larger charge transfer and binding energy. The second factor that stabilizes isomer **1** is its electronic structure, with signatures of metal aromaticity. Both factors act cooperatively, leading to the stabilization of this unusual cage structure. In comparison to the compact bare system, isomer **4**, the mean Pt‐Pt distance is slightly increased in the deuterides, however, the coordination number of the platinum cores are significantly reduced (Table S7). This is in contrast to what has been found in the structural investigations for larger particles on supports.

The experimental scattering data indicate a low proportion of isomer **2** in the studied ensemble. While isomer **1** represents a stable, largely static structure, the opposite applies to isomer **2**. The small energy differences in combination with the low transition barriers of the different deuterium configurations of **2** point to a dynamic and, in combination with the free fcc pocket of the Pt core, a possibly reactive structure.

The void in the cuboctahedral cage structure **1** can be filled maintaining the high symmetry of the cluster forming $O_{h}-{\mathrm{Pt}}_{13}H_{24}$ as suggested by Mager‐Maury et al.^[8]^ and confirmed by preliminary TIED experiments.

## Conflict of Interests

The authors declare no conflict of interest.

1

## Supporting information

As a service to our authors and readers, this journal provides supporting information supplied by the authors. Such materials are peer reviewed and may be re‐organized for online delivery, but are not copy‐edited or typeset. Technical support issues arising from supporting information (other than missing files) should be addressed to the authors.

Supporting Information

## Data Availability

The data that support the findings of this study are available in the supplementary material of this article.
