# Supplementary material for: Pt12H24 −: A Cuboctahedral Platinum Hydride Cluster Cage
Source: Chemphyschem. 2024 Oct 28;25(23):e202400649. doi: 10.1002/cphc.202400649 (PMC11614364; doi:10.1002/cphc.202400649)
Supplement: Supplementary file 1 — Supporting Information [file CPHC-25-e202400649-s001.pdf]

ChemPhysChem

Supporting Information

$Pt_{12}H_{24}^{-}$: A Cuboctahedral Platinum Hydride Cluster Cage

Siddhi Gojare, Dennis Bumüller, Stephan Kohaut, Manuel Kraft, Ulrich Heiz,
Manfred M. Kappes, Karin Fink, and Detlef Schooss*

Supporting Information: $Pt_{12}H_{24}^-$: a Cuboctahedral Platinum Hydride Cluster Cage

Siddhi Gojare,[†] Dennis Bumüller,[†] Stephan Kohaut,[†] Manuel Kraft,[†] Ulrich Heiz,[‡] Manfred M. Kappes,[¶,†] Karin Fink,[†] and Detlef Schooss*[,†]

†*Institute of Nanotechnology, Karlsruhe Institute of Technology (KIT), Kaiserstraße 12, 76131, Germany*

‡*Chair of Physical Chemistry and Catalysis Research Center, Department of Chemistry, School of Natural Sciences, Technical University of Munich, 85748 Garching, Germany*

¶*Institute of Physical Chemistry, Karlsruhe Institute of Technology (KIT), Kaiserstraße 12, 76131, Germany*

E-mail: detlef.schooss@kit.edu

Contents

1 Methods **S-2**

1.1 Trapped Ion Electron Diffraction S-2

1.2 Mass spectrum Platinum Hydrides S-4

1.3 Density functional computations S-4

2 Properties of different isomers **S-6**

3 Cartesian coordinates of isomers **S-16**

References **S-40**

1 Methods

1.1 Trapped Ion Electron Diffraction

Bare platinum clusters were generated in a temperature controlled ($\approx$ 90 K) magnetron sputter source. Hydrides were produced by mixing deuterium (0.3 vol%) into the sputter gas (He and Ar) at a total pressure of 500 Pa. The mass distribution of the cluster ions was analyzed by a time-of-flight spectrometer with a mass resolution of $m/\Delta m \approx 200$ (see Fig.S1). Cluster anions were isolated to a single Pt core size by a quadrupole mass filter, captured in a cooled (95 ± 5 K) quadrupole ion trap and thermalized by collisions with about 1 Pa He for several seconds. A 40 keV electron beam crossed the ion cloud consisting of ca. 10^5 to 10^6 clusters. Electrons scattered during exposure periods of 20–30 s were detected on a phosphor screen assembly and integrated on an external charge-coupled device (CCD) camera.

The total scattering function $I_{\mathrm{tot}}(s)$ as function of the electron momentum transfer s was extracted by radially averaging the sum of several hundred background corrected diffraction pictures. After taking the atomic scattering intensity I_{at} [S1] and an additional unspecific

polynomial based background I_{back} into account, the experimental reduced molecular scattering function $sM^{\text{exp}}(s) = s(I_{\text{tot}} - I_{\text{at}} - I_{\text{back}})/I_{\text{at}}$ was calculated. In order to compare these results with candidate structures, the theoretical reduced molecular scattering function was approximated by

$$sM^{\text{theo}}(s) = S_{\text{c}} \sum_{i}^{N} \sum_{j \neq i}^{N} \exp\left(- \frac{l_{ij}^2}{2} s^2 \right) |f_i(s)||f_j(s)| \cos(\eta_i - \eta_j) \frac{\sin(s k_{\text{s}} r_{ij})}{k_{\text{s}} r_{ij}}, \tag{1}$$

where N is the number of atoms in the cluster, S_{c} and k_{s} are scaling factors for the amplitudes and distances, η_i and f_i are the phase and scattering amplitude of the elastic scattering factors.[S1] r_{ij} is the inter atomic distance and l_{ij} the mean vibrational amplitude. For the latter, a single (averaged) value for every atom-pair type (Pt-Pt, Pt-D or D-D) has been used. Please note that the scattering amplitude of platinum is about 20 times larger than the one of deuterium.[S2] Consequently, the scattering function is largely dominated by the Pt core and variations of the deuterium coverage can be only observed indirectly by changes induced in the platinum core structure.

Experimental broadening was considered by a convolution of sM^{theo} with a Gaussian ($\sigma = 0.15$ Å^{-1}), beam attenuation by the finite trap opening was taken into account by weighting of sM^{theo} with an error function centered at the maximum scattering angle ($s = 14$ Å^{-1}). For comparison of theoretical and experimental data, a χ^2-fit was performed minimizing the weighted differences by variation of S_c, k_s, $l_{\text{Pt-Pt}}$, $l_{\text{Pt-D}}$, $l_{\text{D-D}}$ and the parameters of I_{back}. The level of agreement between the experiment and model structure was determined by a weighted profile factor[S3] given by

$$R_{\text{w}} = \sqrt{\sum_k w_k (sM_k^{\text{theo}} - sM_k^{\text{exp}})^2 / \sum_k w_k (sM_k^{\text{exp}})^2}. \tag{2}$$

The weighting factors w_k were calculated from the error propagated standard deviation of the experimental data.

1.2 Mass spectrum Platinum Hydrides

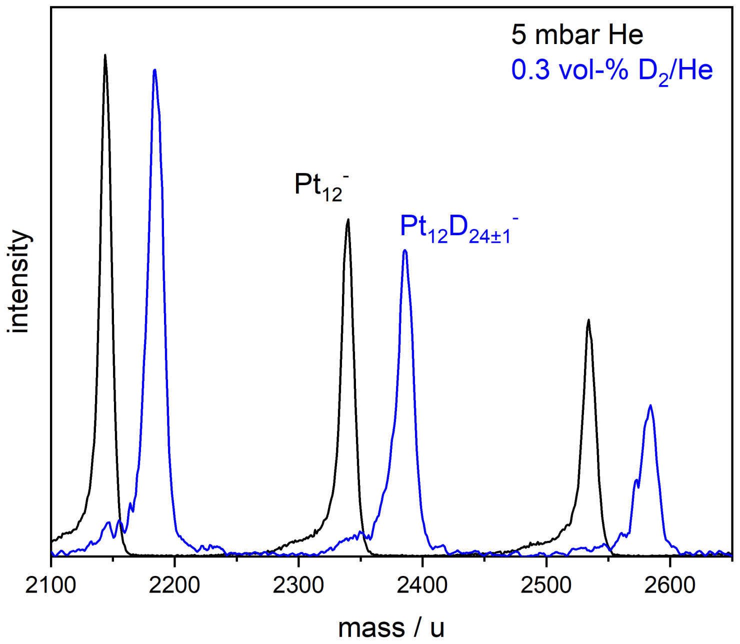

Figure S1: Typical mass spectrum of bare and deuterated Pt-cluster anions around Pt_{12}^-. The mean value and width of the number of deuterium atoms was obtained by fitting an empirical asymmetric peak function (Gram-Charlier) to the mass spectral profiles for the bare and D-loaded mass peaks. The formal mean stoichiometry determined in this way was $Pt_{12}D_{23.7}$ with a FWHM of 1.6 D.

1.3 Density functional computations

The best fitting geometries to the experimental molecular scattering data have been re-optimized using density functional theory (DFT) computations within the TURBOMOLE package (version 7.7).[S4,S5] The RI-J (resolution of identity for the Coulomb term J) approximation was used.[S6,S7] In the first step, the cluster geometries were re-optimized using the BP86[S8,S9] in combination with the def2-SVP basis set and the TPSS[S10] functional in combination with the def2-TZVP basis set (TPSS/def2-TZVP).[S11] For Pt, 60 core electrons were replaced by a scalar relativistic effective core potential.[S12] All geometry optimizations were

performed without any symmetry constraints. Fermi smearing was used to enable fractional occupations to eventually get electronic states without any holes. The initial temperature was fixed to T=300K and was self-consistently lowered to T=10K. Vibrational frequencies of all cluster structures were analyzed to verify that the final structures are local minima. Different spin multiplicities were checked. For charged clusters a doublet spin state and for neutral clusters a singlet spin state was found to be the most stable one in all cases.

For the heavy elements such as Pt, the effect of spin-orbit coupling (SOC) needs to be taken into account to obtain an accurate ground state structure.[S13,S14] The previous study on bare Pt_n^- clusters showed that SOC contribution had a significant influence on the relative energies and the geometry of the isomers.[S15] Therefore, the final geometries were re-optimized using a relativistic two-component method[S16] with the TPSS functional in combination with the dhf-TZVP-2c basis set (2C-TPSS/dhf-TZVP-2c).

The effect of dispersion correction was evaluated using DFT-D4.[S17] All relative energies were corrected by the zero-point energies. Free energy computations were carried out considering an experimental temperature of 120K and pressure of 0.44 mbar. The computation of the activation barriers and corresponding transition states for the isomer transformation were carried out using the Nudged Elastic Band (NEB) method.[S18] The d-band center of all the isomers were calculated using Mulliken population of the d-orbitals $n(\epsilon)$ using

$$\epsilon_{\mathrm{d}} = \frac{\sum_\alpha \sum_\epsilon \epsilon n(\epsilon)}{\sum_\alpha \sum_\epsilon n(\epsilon)}, \tag{3}$$

where ϵ is the molecular orbital energy and α goes over the selected surface Pt atoms only. A natural population analysis[S19] and the Wiberg bond indices[S20] were computed to predict the charge distribution and the nature of the chemical bonds. To test for spherical aromaticity, the Nucleus Independent Chemical Shift (NICS) value was calculated.[S21] In the density of states plots, the energy of the highest occupied orbital was chosen as Fermi level. The orbital energies were broadened by Gaussians (0.272 eV FWHM).

2 Properties of different isomers

Table S1: Relative electronic energies E (TPSS/def2-TZVP) and profile factors R_w of the isomers for different deuterium distributions generated using isomer 1 core.

isomer	E/eV	R_w %
**1**	0	5.5
1-1	1.14	4.8
1-2	1.35	4.5
1-3	1.40	5.4

Table S2: Relative electronic energies E (TPSS/def2-TZVP) and profile factors R_w for different deuterium distributions generated using isomer 2 core.

isomer	E/eV	R_w/%
**2**	0	12
2-1	0.40	12
2-2	0.46	11
2-3	0.54	12
2-4	0.55	10
2-5	0.60	13
2-6	0.60	11
2-7	0.74	13
2-8	0.77	14
2-9	0.82	11

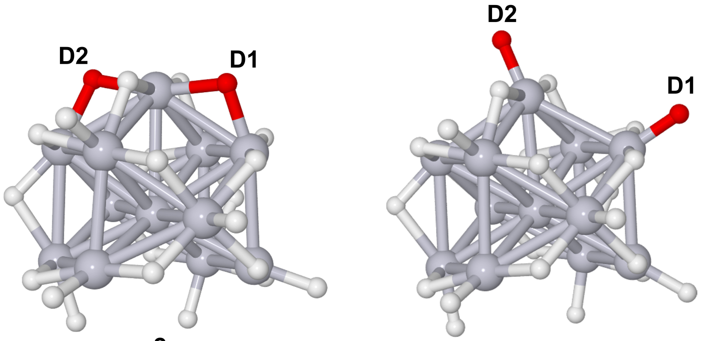

2 2.1

Figure S2: Isomer **2** core structure with different distributions of deuterium atoms. The isomer on the left-hand side is the most stable one.

Table S3: Comparison of relative energy E (TPSS/def2-TZVP) for different charge states of the isomers 1-3. All values are zero point energy corrected (values in the bracket denote the relative contribution of ZPE).

isomer	E/eV $Pt_{12}D_{24}^{-}$	E/eV $Pt_{12}D_{24}$	E/eV $Pt_{12}D_{24}^{+}$	E/eV $Pt_{12}D_{24}^{2-}$
1	0	0	0.44 (-0.005)	0
2	0.72 (-0.052)	0.31 (0.03)	0	1.2 (-0.144)
3	0.99 (-0.072)	1.05 (-0.08)	1.41 (-0.118)	0.94 (-0.063)

Table S4: Comparison of the relative energies of isomers 1-3 for TPSS/def2-TZVP, 2C-TPSS/dhf-TZVP-2c, the dispersion corrections, and relative of the relative difference of zero point energy and free Gibbs energy (G). The contribution of the relative zero point energy is given in parenthesis.

isomer	TPSS/def2-TZVP	2C-TPSS/dhf-TZVP-2c	disp. corr./eV (2C)	G/eV (2C)
1	0	0	-5.03	0
2	0.72 (-0.052)	0.52 (-0.053)	-5.05	0.003
3	0.99 (-0.071)	0.96 (-0.018)	-5.00	-0.006

Table S5: Calculated adsorption energies (electronic energies) of differently bound deuterium atoms of isomer 2 ($Pt_{12}D_{24}^{-}$→$Pt_{12}D_{23}^{-}$+$\frac{1}{2}D_2$) at TPSS/def2-TZVP level of theory. For positions, see Fig. S9.

bridge D_x atom	adsorption energy (eV)	on-top D_x atom	adsorption energy (eV)
1	1.22	19	0.55
2	1.19	20	0.47
3	1.14	21	0.43
4	1.07	22	0.42
5	0.98	23	0.26
6	0.92	24	0.23
7	0.91		
8	0.8		
9	0.79		
10	0.72		
11	0.71		
12	0.7		
13	0.64		
14	0.61		
15	0.47		
16	0.38		
17	0.37		
18	0.33		

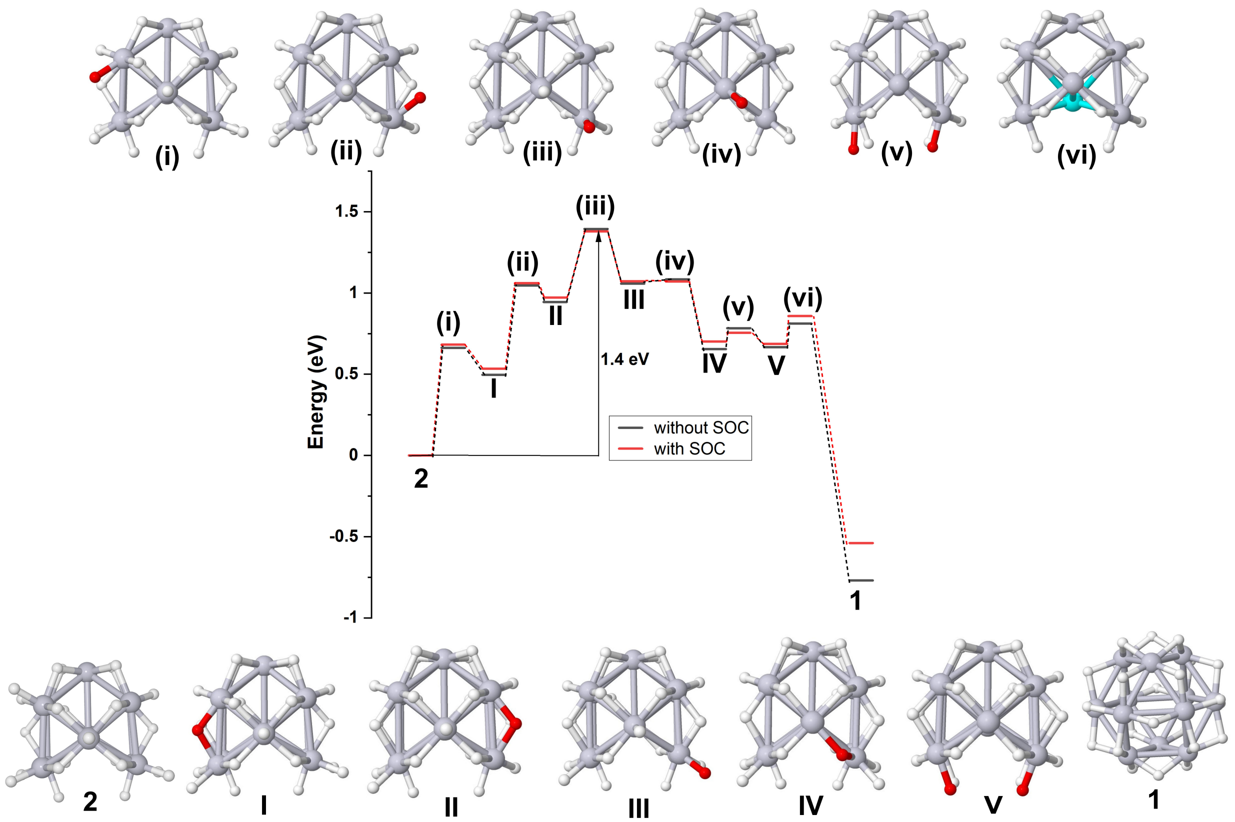

Figure S3: A reaction pathway describing the conversion of isomer **2** to **1** with and without the inclusion of spin-orbit coupling effect. The deuterium atoms in red color denote the ones whose position are changed. Note that electronic energies are given relative to isomer **2**.

Table S6: Calculated charges using natural population analysis of bare Pt_{12}^-. For positions, see Figure S8

number	atom	charge
1	Pt	-0.197
2	Pt	-0.197
3	Pt	-0.197
4	Pt	-0.065
5	Pt	-0.065
6	Pt	-0.065
7	Pt	-0.065
8	Pt	-0.065
9	Pt	-0.065
10	Pt	-0.008
11	Pt	-0.008
12	Pt	-0.008

Table S7: Mean bond lengths (TPSS/def2-TZVP) and mean coordination number CN of the platinum core

isomer	$d_{\mathrm{Pt-Pt}}$/pm	$d_{\mathrm{Pt-D}}$/pm		CN
		bridge	on-top	
**1**	0.271	0.176	–	4.0
**2**	0.277	0.176	0.158	5.16
**3**	0.272	0.176	–	4.0
**4**	0.268	-	-	5.5

Table S8: Calculated charges using natural population analysis of isomer 2. For positions, see Figure S9.

number	atom	charge	D site
1	D	-0.104	bridge
2	D	-0.092	bridge
3	D	-0.107	bridge
4	D	-0.111	bridge
5	D	-0.1	bridge
6	D	-0.081	bridge
7	D	-0.068	bridge
8	D	-0.108	bridge
9	D	-0.077	bridge
10	D	-0.043	bridge
11	D	-0.116	bridge
12	D	-0.076	bridge
13	D	-0.105	bridge
14	D	-0.113	bridge
15	D	-0.103	bridge
16	D	-0.069	bridge
17	D	-0.127	bridge
18	D	-0.115	bridge
19	D	-0.042	on-top
20	D	-0.04	on-top
21	D	-0.048	on-top
22	D	-0.032	on-top
23	D	-0.068	on-top
24	D	-0.044	on-top
25	Pt	-0.388	
26	Pt	0.094	
27	Pt	0.043	
28	Pt	0.131	
29	Pt	0.155	
30	Pt	0.209	
31	Pt	0.089	
32	Pt	0.122	
33	Pt	0.156	
34	Pt	0.217	
35	Pt	0.081	
36	Pt	0.078	

Table S9: Calculated charges using natural population analysis of isomer 1.All other charges are similar to the ones given in the table

Number	Atoms	Charge
1	Pt	0.12641
3	D	-0.10533

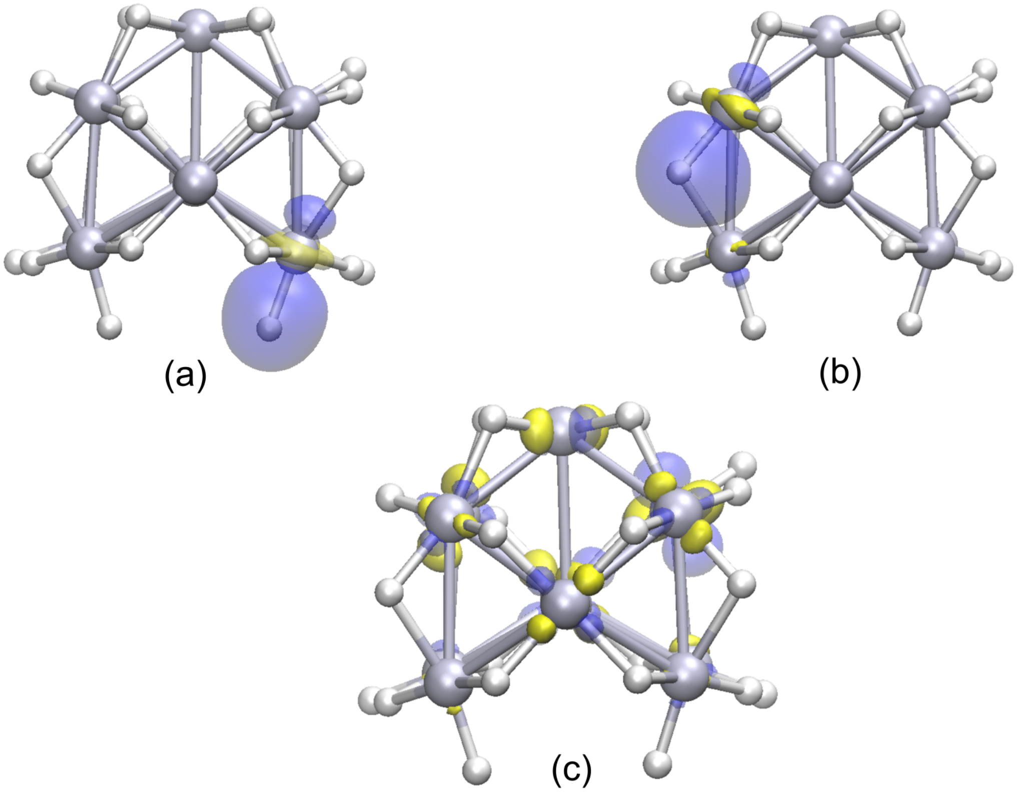

Figure S4: Localized orbitals of isomer **2** obtained by Boys localization. (a) a typical 2-center - 2 electron bond between Pt-D, and (b) a 3-center - 2 electron bond Pt-D-Pt bond. (c) typical d-orbitals at different Pt atoms.

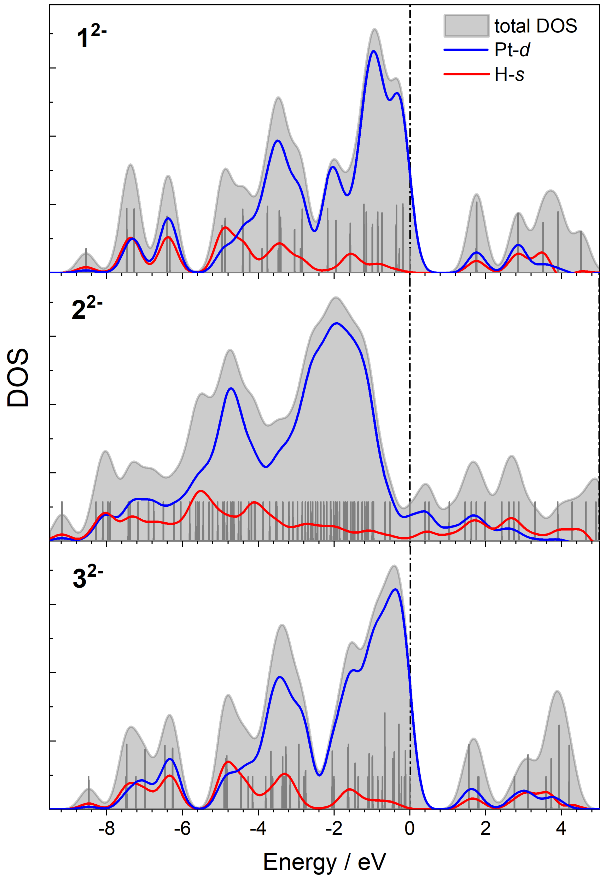

Figure S5: DOS for the dianions $Pt_{12}D_{24}^{2-}$ of isomer **1**-**3**.

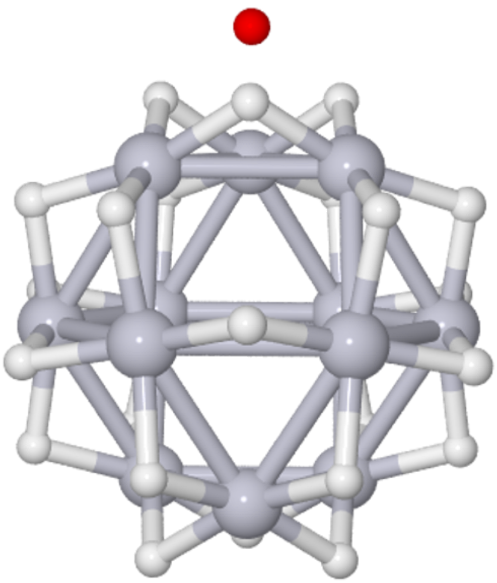

Figure S6: Isomer **1** with the position of the pseudo atom in red at which the NICS value was calculated.

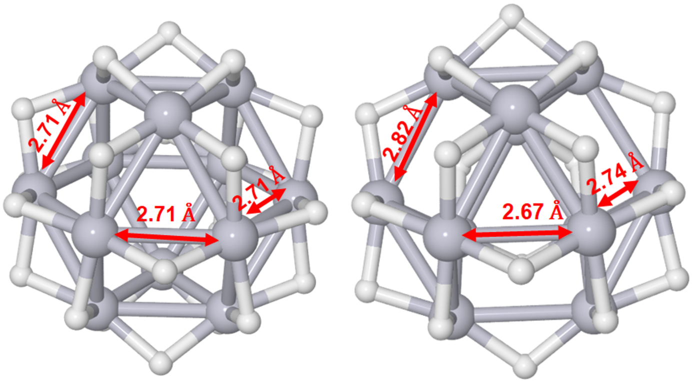

Figure S7: Pt-Pt bond lengths in isomer **1** and **3**.

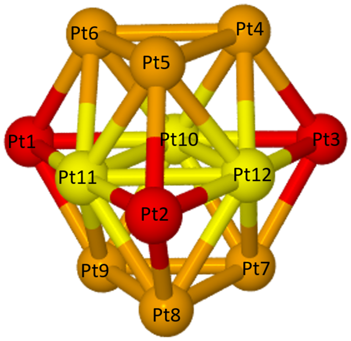

Figure S8: Structure and charge distribution of Pt_{12}^{-} (red - high, orange - medium, yellow - low charge.)

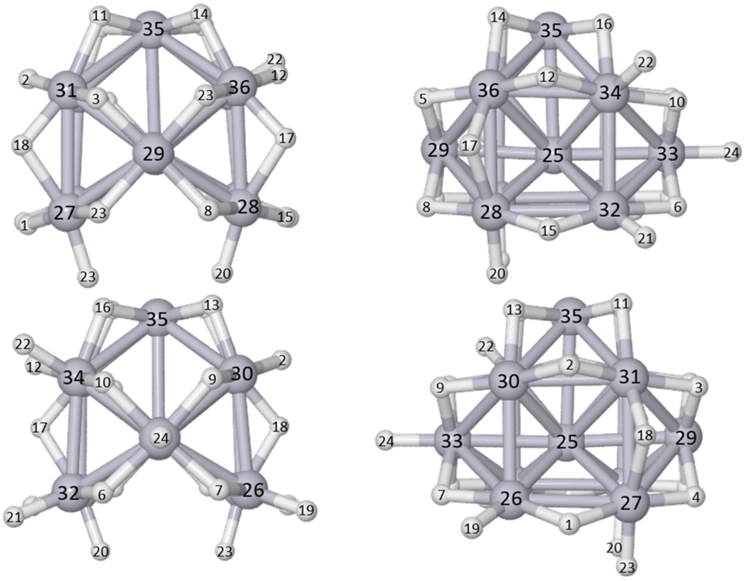

Figure S9: Different views of isomer **2**.

3 Cartesian coordinates of isomers

energies are given in Hartree

Isomer 1:(method: TPSS/dhf-TZVP-2c)

Energy= -1450.40223

Pt	1.4457026	-1.6268444	1.6188080
Pt	-1.2519436	-1.5028786	1.8809997
H	-2.8427640	-0.7566915	1.7790444
H	-1.7629828	-2.8280782	0.8416202
H	1.5357436	-0.5655225	3.0202580
Pt	0.2827237	0.6061906	2.6301386
H	-0.9717175	-0.4521343	3.2662800
H	3.0497111	-1.0252534	1.2092673
Pt	2.6982888	-0.1242100	-0.2609531
H	3.1044505	1.3179714	0.6629431
Pt	1.5342947	2.1084421	0.7488053
H	1.5917095	1.7729459	2.4759141
Pt	-2.6981044	0.1246199	0.2610342
H	-3.0502081	1.0271342	-1.2084527
H	-2.7860187	1.5875960	1.2356043
H	-0.9187270	1.8914469	2.7200500
Pt	-1.1635489	2.2336381	1.0106690
H	0.2450759	3.2868019	0.9727016
Pt	1.1636928	-2.2334586	-1.0102329
H	1.6199605	-2.9850740	0.5139799
H	-0.2450735	-3.2869258	-0.9716434
H	2.7861017	-1.5880662	-1.2349754

H	1.7616569	2.8283448	-0.8413966
Pt	-1.5340843	-2.1084862	-0.7488924
H	-3.1036955	-1.3169252	-0.6645741
H	-1.5901179	-1.7747247	-2.4764226
H	0.1088992	-2.5275353	2.3232949
Pt	-0.2826002	-0.6063777	-2.6302109
H	2.8425037	0.7556538	-1.7793444
H	0.9711288	0.4529319	-3.2669583
H	0.9195033	-1.8903099	-2.7200789
H	-1.6191581	2.9851424	-0.5142102
Pt	-1.4460699	1.6267587	-1.6186213
Pt	1.2516660	1.5026585	-1.8813807
H	-0.1087066	2.5269074	-2.3241374
H	-1.5372922	0.5643124	-3.0189273

Isomer 2:(method: TPSS/dhf-TZVP-2c)

Energy= -1450.37504

Pt	0.1878900	-0.1302391	0.2939932
Pt	-1.3057896	-1.1240102	2.3270471
H	-2.3369556	-1.5798069	3.4355527
H	-1.9043019	-2.5123395	1.4436460
H	1.4400655	-0.1908425	3.5357113
Pt	0.4648153	0.8764716	2.8881617
H	-0.8533135	-0.0325480	3.6201409
H	3.2233122	-1.2217924	1.2182411
Pt	2.8684889	-0.4138737	-0.0908050
H	3.3428474	1.0173946	0.7747356

Pt	1.8611827	2.0301707	0.7798269
H	1.8920153	1.8146136	2.5644065
Pt	-2.5046925	0.3672855	0.4269250
H	-2.8906653	1.2131471	-1.0263960
H	-2.4965224	1.9689278	1.2630372
H	-0.7002521	2.3476455	2.6437232
Pt	-0.8752450	2.4785020	0.9941670
H	0.6834551	3.3228582	0.9104118
Pt	1.1657775	-2.4533583	-0.7014852
H	-0.3236836	-3.3644834	-0.4551471
H	1.6869267	-3.7409407	-1.4581241
H	2.8178787	-1.9683787	-0.9510306
H	2.1370526	2.5659080	-0.8664296
Pt	-1.5729108	-2.1448321	-0.2523640
H	-2.6040842	-3.3176134	-0.5568416
H	-1.5468535	-1.9234848	-2.0267675
H	-3.0423478	-1.1301716	-0.2610594
Pt	-0.2479293	-0.8324162	-2.3235960
H	2.9651443	0.4950542	-1.7846782
H	1.1103668	0.0959147	-3.0822866
H	-0.3788964	-1.3593395	-3.8140314
H	-1.3117967	3.1198103	-0.6908970
Pt	-1.2199877	1.6834966	-1.6088138
Pt	1.4916667	1.2590885	-1.8732867
H	0.0991159	2.3760996	-2.4179602
H	-1.3217737	0.4080824	-2.8777273

Isomer 3:(method: TPSS/dhf-TZVP-2c)

Energy= -1450.36637

H	2.9429853	1.6992574	0.0000000
H	1.2465619	0.7189822	3.1647010
H	1.2465619	0.7189822	-3.1647010
H	-1.3586149	2.5894866	-1.7414320
H	1.3586149	2.5894866	-1.7414320
Pt	0.0000000	1.5428294	-2.2442096
H	-2.9203993	-0.1143362	-1.7413555
H	1.3586149	2.5894866	1.7414320
Pt	1.3202525	2.3893205	0.0000000
H	0.0000000	3.5419377	0.0000000
Pt	-1.3202525	2.3893205	0.0000000
Pt	0.0000000	1.5428294	2.2442096
H	-1.3586149	2.5894866	1.7414320
H	-2.9429853	1.6992574	0.0000000
H	-2.9203993	-0.1143362	1.7413555
Pt	-2.7293677	-0.0517249	0.0000000
H	-1.2465619	0.7189822	3.1647010
H	-1.2465619	0.7189822	-3.1647010
H	-0.0000000	-1.4401041	3.1647820
Pt	2.7293677	-0.0517249	0.0000000
H	2.9203993	-0.1143363	-1.7413555
H	1.5570929	-2.4728803	-1.7415209
Pt	1.3352290	-0.7716386	-2.2441164
H	-0.0000000	-1.4401041	-3.1647820
Pt	-1.4084988	-2.3388279	0.0000000

H	3.0677405	-1.7716279	0.0000000
H	-0.0000000	-3.3986371	0.0000000
H	-1.5570928	-2.4728803	-1.7415209
Pt	1.4084988	-2.3388279	0.0000000
H	-1.5570928	-2.4728803	1.7415209
H	1.5570929	-2.4728803	1.7415209
Pt	1.3352290	-0.7716386	2.2441164
H	2.9203993	-0.1143363	1.7413555
Pt	-1.3352290	-0.7716386	2.2441164
Pt	-1.3352290	-0.7716386	-2.2441164
H	-3.0677405	-1.7716279	0.0000000

Isomer 4 (bare Pt_{12}^{-}):(method: TPSS/def2-TZVP)

Energy= -1432.5612

Pt	-0.0328040	-0.0000472	3.0249376
Pt	0.0077873	-2.1347910	1.5287267
Pt	0.0077107	2.1344719	1.5284569
Pt	1.3842984	-0.0000761	0.7562247
Pt	1.3889689	-2.1959953	-0.7979263
Pt	-1.3165963	-2.1351698	-0.7682103
Pt	2.6697742	0.0000421	-1.5395890
Pt	-1.5903852	0.0001201	0.9182144
Pt	1.3888581	2.1958107	-0.7977578
Pt	-1.3165153	2.1349302	-0.7682978
Pt	0.0407512	0.0000241	-1.5712640
Pt	-2.6316040	-0.0000927	-1.4858558

Isomers generated using isomer 1 core:

Isomer 1-1:(method: TPSS/def2-TZVP)

Energy = -1447.228224995

Pt	1.4744186	-1.5143661	1.7267028
Pt	-1.2442052	-1.3446398	1.9323312
H	-2.7705537	-0.4901583	1.8611540
H	-1.8805029	-2.6479787	0.9216466
H	1.5805941	-0.4357495	3.1307923
Pt	0.3244598	0.7308624	2.7083958
H	-0.9258425	-0.3158848	3.3208874
H	3.0672911	-0.9106160	1.2920975
Pt	2.7166256	0.0183525	-0.1747462
H	3.1217499	1.4514804	0.7735442
Pt	1.5699738	2.2831660	0.8487114
H	1.6112461	1.9329079	2.5749462
Pt	-2.6562297	0.3131697	0.3007585
H	-3.0678382	1.2299239	-1.1520021
H	-2.2820092	-3.3723786	-1.2366475
H	-0.9351218	1.9445850	2.7150460
Pt	-1.0785718	2.2759886	0.9923830
H	0.2567625	3.4502224	1.0924452
Pt	1.0877472	-2.0237071	-0.8948484
H	1.6002944	-2.8319538	0.5995790
H	-0.2093836	-3.0831557	-0.8312848
H	2.7523471	-1.3895846	-1.1660203
H	1.7683046	2.9648273	-0.7666892

Pt	-1.7173709	-2.0132338	-0.7176462
H	-3.2524093	-1.4630937	-0.6524228
H	-1.7111912	-1.6032801	-2.4181209
H	0.1088412	-2.3971120	2.3873572
Pt	-0.3528312	-0.4596738	-2.5732259
H	2.8262679	0.8878032	-1.6819715
H	0.9297499	0.5758249	-3.1528978
H	0.8312019	-1.7567509	-2.6100576
H	-1.6281911	3.1260041	-0.4697523
Pt	-1.4815995	1.8211074	-1.6072604
Pt	1.2242747	1.6307384	-1.7797158
H	-0.0970247	2.6783880	-2.2803463
H	-1.5612738	0.7379652	-3.0131223

Isomer 1-2:(method: TPSS/def2-TZVP)

Energy = -1447.220861396

Pt	1.4404043	-1.7100341	1.7438668
Pt	-1.2745595	-1.6027887	1.9815092
H	-2.7529779	-0.7475113	1.8781766
H	-1.8109569	-2.9063146	0.9035964
H	1.4904533	-0.6134763	3.1163583
Pt	0.2148014	0.5409742	2.7398241
H	-0.9953429	-0.5713719	3.3683020
H	3.0240978	-1.0724756	1.3242938
Pt	2.6832902	-0.1742160	-0.1416475
H	3.0651307	1.2208318	0.7768134
Pt	1.4502538	2.0532768	0.8415887

H	1.4788844	1.7344556	2.5803706
Pt	-2.5320146	0.1310406	0.3349823
H	-3.0180227	1.0673981	-1.0410011
H	-2.2143294	2.6836429	-2.4305411
H	-1.0422715	1.7645242	2.8362884
Pt	-1.2209596	2.2852216	1.1721467
H	0.4479402	3.3361894	1.1268227
Pt	1.0999131	-2.2588903	-0.9019367
H	1.5957196	-3.0318700	0.6134189
H	-0.3114241	-3.3531693	-0.9247631
H	2.6995190	-1.6706127	-1.1399535
H	1.7518472	2.7943222	-0.7342657
Pt	-1.5999991	-2.2018619	-0.6823346
H	-3.1274510	-1.2705143	-0.5254534
H	-1.6366166	-1.7800871	-2.3862630
H	0.1113862	-2.6269672	2.4184572
Pt	-0.3253099	-0.6117599	-2.5410164
H	2.8277458	0.6928400	-1.6569285
H	0.9619953	0.4222566	-3.1709015
H	0.8675563	-1.9038281	-2.5900402
H	-1.5992994	3.0320842	-0.3703399
Pt	-1.4359910	1.7091160	-1.5296079
Pt	1.2870614	1.5027092	-1.8283304
H	-0.0660913	2.5488589	-2.2531893
H	-1.5343825	0.5880073	-2.9083021

Isomer 1-3:(method: TPSS/def2-TZVP)

Energy = -1447.218998125

Pt	1.3226429	-1.5278897	1.6085498
Pt	-1.4205292	-1.4002035	1.8895426
H	-2.9678353	-0.5856970	1.7333840
H	-1.9201916	-2.7030094	0.8226044
H	1.3821267	-0.4332983	2.9953742
Pt	0.1243645	0.7350792	2.6428899
H	-1.1293904	-0.3350739	3.2622667
H	2.9192145	-0.8884193	1.1604791
Pt	2.6057310	-0.0125747	-0.3117930
H	2.9701839	1.4203210	0.6164960
Pt	1.3771587	2.2127220	0.7175781
H	1.4208183	1.9035705	2.4489762
Pt	-2.8156125	0.2266146	0.1981683
H	-3.1941365	1.1533727	-1.2504847
H	2.1535753	-2.4499738	2.5138384
H	-1.1073335	1.9879927	2.6446322
Pt	-1.2861328	2.3504194	0.9486908
H	0.1745170	3.4239577	0.9661476
Pt	1.0527094	-2.1536044	-1.0679381
H	1.4775019	-2.8729273	0.4600760
H	-0.3627057	-3.1770765	-1.0080564
H	2.6586578	-1.4714959	-1.2986102
H	1.6030729	2.9385862	-0.8736362
Pt	-1.6682865	-1.9910566	-0.7660910
H	-3.2449345	-1.2825549	-0.6969938
H	-1.7323691	-1.6556079	-2.4952786

H	-0.0446936	-2.4073166	2.2998092
Pt	-0.4292026	-0.4971866	-2.6753449
H	2.6846283	0.8712234	-1.8283556
H	0.8191649	0.5704508	-3.3021233
H	0.7779215	-1.7582828	-2.7581596
H	-1.7772838	3.1035811	-0.5649817
Pt	-1.6048367	1.7595511	-1.6771829
Pt	1.1129082	1.6272312	-1.9274283
H	-0.2513184	2.6432909	-2.3678595
H	-1.6801047	0.6752845	-3.0591862

Isomers generated using isomer 2 core:

Isomer 2-1:(method: TPSS/def2-TZVP)

Energy = -1447.234196247

Pt	0.1581452	-0.0498831	0.2252664
Pt	-1.2672272	-1.1679421	2.2376765
H	-2.0600088	-1.8414627	3.4302226
H	-1.8923421	-2.5182232	1.3284937
H	1.4287330	-0.2196993	3.5153112
Pt	0.4662872	0.8352392	2.8385940
H	-0.8462796	-0.0984933	3.5522676
H	3.4232347	-1.2743243	1.2320605
Pt	2.9233900	-0.4484085	-0.0081620
H	3.4095250	0.9922137	0.8594999
Pt	1.9461198	2.0068076	0.8217012
H	1.8988773	1.8066198	2.5885405

Pt	-2.5316249	0.4423504	0.3597952
H	-4.0849967	0.7568636	0.4280535
H	-2.4888544	2.0378123	1.0920005
H	-0.6928857	2.3052115	2.5816260
Pt	-0.7976557	2.5079668	0.9262012
H	0.7694435	3.3183131	0.9180398
Pt	1.1627779	-2.4202021	-0.6736262
H	-0.3381175	-3.3045306	-0.5535556
H	1.7052825	-3.7410064	-1.3525153
H	2.8566375	-1.9882927	-0.8507120
H	2.2847744	2.5344235	-0.8250742
Pt	-1.5888316	-2.0677117	-0.3606844
H	-2.5908238	-3.2500722	-0.6996339
H	-1.5222140	-1.8225266	-2.1206004
H	-3.0654454	-1.0532050	-0.3263383
Pt	-0.1371299	-0.8029571	-2.3844477
H	3.1315370	0.5174223	-1.7325167
H	1.2841866	0.0657613	-3.0558600
H	-0.2086882	-1.3452537	-3.8729666
H	-1.1003406	3.1279938	-0.7704338
Pt	-1.0110575	1.7006119	-1.6896243
Pt	1.6548276	1.2122090	-1.8110632
H	-1.1399397	2.7464360	-2.8515620
H	-1.1393161	0.4999388	-2.9959739

Isomer 2-2:(method: TPSS/def2-TZVP)

Energy = -1447.219948295

Pt	0.0467254	-0.0172697	0.2184013
Pt	-1.3676380	-1.1436132	2.2877278
H	-2.2368181	-1.7412491	3.4635680
H	-2.0535020	-2.4424414	1.3512013
H	1.3982565	-0.0613131	3.4742015
Pt	0.3635860	0.9655657	2.8565845
H	-0.8574101	-0.0786703	3.5889174
H	3.1222947	-1.1283854	1.1852554
Pt	2.7833649	-0.3258819	-0.1357058
H	3.2253091	1.1110623	0.7364590
Pt	1.7458914	2.1278232	0.7331833
H	1.7510726	1.9448278	2.5213427
Pt	-2.6290038	0.4768310	0.3075273
H	0.2465989	-1.2819004	1.2782165
H	-2.6640913	2.0668516	1.1059031
H	-0.8759194	2.3624622	2.6008298
Pt	-1.0077653	2.5576161	0.9392606
H	0.5525534	3.4040683	0.8805363
Pt	1.0713380	-2.3788264	-0.7531782
H	-0.4374886	-3.2672994	-0.5360540
H	1.5850691	-3.6421889	-1.5330129
H	2.7352109	-1.8853995	-0.9690666
H	2.0269214	2.6518558	-0.9106021
Pt	-1.7156637	-2.0532990	-0.3639500
H	-2.7314045	-3.2189231	-0.6845151
H	-1.6430203	-1.8207627	-2.1282478
H	-3.1564367	-1.0013627	-0.4436661

Pt	-0.3107035	-0.7697614	-2.4240655
H	2.9278509	0.5840161	-1.8230229
H	1.0857770	0.1521318	-3.1420842
H	-0.4529923	-1.2671948	-3.9125418
H	-1.3759868	3.1624122	-0.7171381
Pt	-1.2484588	1.7103338	-1.6388298
Pt	1.4467417	1.3267332	-1.9437605
H	0.0261186	2.4103736	-2.5277360
H	-1.3763772	0.5107778	-2.9419384

Isomer 2-3:(method: TPSS/def2-TZVP)

Energy = -1447.221952128

Pt	0.0946018	0.0453108	0.0781824
Pt	-1.3138831	-1.1907528	2.0537343
H	-2.0042143	-1.9662195	3.2582954
H	-2.0807561	-2.4272064	1.0906745
H	1.4575154	-0.2863543	3.1526152
Pt	0.4393039	0.8190117	2.6749882
H	-0.7842153	-0.2244027	3.4071990
H	4.3005040	-0.8209835	0.3732418
Pt	2.7613347	-0.4731907	0.1899980
H	3.3305849	1.0658665	0.7924030
Pt	1.8893557	2.0917408	0.7307098
H	1.8165257	1.8940060	2.4819708
Pt	-2.6361756	0.5995758	0.1663555
H	-2.6969384	-0.1381294	1.6843098
H	-2.5594854	2.1911369	0.9383045

H	-0.7655835	2.2677088	2.4844289
Pt	-0.8570712	2.5865263	0.8212334
H	0.7030196	3.4061976	0.8150016
Pt	0.9973331	-2.4452785	-0.4349674
H	-0.5381266	-3.2665775	-0.5548900
H	1.5440087	-3.9264530	-0.5275665
H	2.7135940	-2.1635763	-0.2659757
H	2.2475534	2.5777125	-0.9199172
Pt	-1.7122766	-1.9401439	-0.5772337
H	-2.7694144	-3.0499521	-0.9809438
H	-1.5709088	-1.7572441	-2.3147206
H	-3.1270882	-0.8865040	-0.6429599
Pt	-0.1565039	-0.7072265	-2.5807115
H	2.8901730	0.2190739	-1.4256665
H	1.2313875	0.1550528	-3.2309086
H	0.8375223	-2.0016843	-2.2892781
H	-1.0822597	3.2383979	-0.8009113
Pt	-1.1153179	1.7924283	-1.8141542
Pt	1.6288103	1.2508242	-1.9015927
H	-1.8013784	2.7559439	-2.8405315
H	-1.3115308	0.7153649	-3.0907166

Isomer 2.4:(method: TPSS/def2-TZVP)

Energy = -1447.213798196

Pt	0.0677555	-0.2148994	0.3442317
Pt	-1.5615410	-1.1383660	2.3675780
H	-3.0638255	-0.4168828	2.0942840

H	1.4021633	-1.1260058	0.7544476
H	1.1955451	-0.3091332	3.6270675
Pt	0.2840847	0.7925541	2.9388571
H	-1.1019536	-0.0148066	3.6575071
H	3.6627001	-1.2992727	1.0676291
Pt	2.9585743	-0.5287848	-0.1264374
H	3.3390702	0.9197394	0.7663241
Pt	1.7927988	1.8743195	0.8065543
H	1.7852782	1.6321299	2.5759408
Pt	-2.7594530	0.3294605	0.4143162
H	-2.9900069	1.1149409	-1.1045913
H	-2.6573934	1.9307301	1.2049387
H	-0.8186747	2.2806178	2.6450691
Pt	-0.9778010	2.3638534	0.9853884
H	0.6067627	3.1534024	0.9241221
Pt	1.0994016	-2.5426888	-0.7119559
H	-0.4317426	-3.3632039	-0.3920591
H	1.4696700	-3.8198878	-1.5630410
H	2.7803061	-2.0856229	-0.9902538
H	2.0853388	2.4167770	-0.8435386
Pt	-1.7212646	-2.1691957	-0.2418808
H	-2.0758312	-2.5448158	1.4404936
H	-1.6596989	-2.0229249	-2.0104808
H	-3.2228535	-1.1914373	-0.2974977
Pt	-0.3281556	-0.9525649	-2.3000464
H	2.8899983	0.3521340	-1.7941321
H	1.0268159	-0.0462084	-3.0641126

H	-0.4741169	-1.5304290	-3.7730273
H	-1.3740899	2.9840878	-0.6996026
Pt	-1.3029842	1.5414097	-1.6047581
Pt	1.4184546	1.1202545	-1.8445135
H	0.0642929	2.2354452	-2.3771864
H	-1.4076249	0.2752742	-2.8756340

Isomer 2-5:(method: TPSS/def2-TZVP)

Energy = -1447.220073052

Pt	0.1853880	-0.1761830	0.2694418
Pt	-1.3888558	-1.1728288	2.2475752
H	-2.4758306	-1.6204663	3.2976014
H	-1.9662771	-2.5419566	1.3096644
H	1.3664595	-0.3063996	3.5378502
Pt	0.4249926	0.7836432	2.8719273
H	-0.9297121	-0.1003828	3.5631414
H	3.3256943	-1.3405932	1.1187766
Pt	2.9252619	-0.5140833	-0.1703719
H	3.3600027	0.8886356	0.7563435
Pt	1.8952055	1.9318502	0.7840405
H	1.8869414	1.6714117	2.5594930
Pt	-2.5084133	0.3552038	0.3366069
H	-2.8406376	1.2139036	-1.1186700
H	-2.4790726	1.9580711	1.1867822
H	-0.6999116	2.2678597	2.6188825
Pt	-0.8416854	2.4403078	0.9665900
H	0.7318648	3.2490028	0.9227614

Pt	1.2090173	-2.6114583	-0.8285777
H	-0.3447942	-3.4371885	-0.6103926
H	0.8212672	-1.7300501	0.6097422
H	2.8746845	-2.0458602	-1.0525529
H	2.2012562	2.4926426	-0.8502719
Pt	-1.5794083	-2.1891514	-0.3821554
H	-2.6084884	-3.3517475	-0.7178394
H	-1.4887618	-1.9538025	-2.1486481
H	-3.0131224	-1.1274020	-0.4001877
Pt	-0.1677534	-0.8693951	-2.3692885
H	3.0302880	0.4331746	-1.8191592
H	1.1868017	0.0557686	-3.1148004
H	-0.2272221	-1.4225295	-3.8551309
H	-1.2258969	3.1170263	-0.6753105
Pt	-1.1518220	1.6692206	-1.6431234
Pt	1.5615973	1.2127436	-1.8914477
H	0.1971535	2.3493901	-2.4170645
H	-1.2462107	0.4216229	-2.8922277

Isomer 2.6:(method: TPSS/def2-TZVP)

Energy = -1447.242032648

Pt	0.1878900	-0.1302391	0.2939932
Pt	-1.3057896	-1.1240102	2.3270471
H	-2.3369556	-1.5798069	3.4355527
H	-1.9043019	-2.5123395	1.4436460
H	1.4400655	-0.1908425	3.5357113
Pt	0.4648153	0.8764716	2.8881617

H	-0.8533135	-0.0325480	3.6201409
H	3.2233122	-1.2217924	1.2182411
Pt	2.8684889	-0.4138737	-0.0908050
H	3.3428474	1.0173946	0.7747356
Pt	1.8611827	2.0301707	0.7798269
H	1.8920153	1.8146136	2.5644065
Pt	-2.5046925	0.3672855	0.4269250
H	-2.8906653	1.2131471	-1.0263960
H	-2.4965224	1.9689278	1.2630372
H	-0.7002521	2.3476455	2.6437232
Pt	-0.8752450	2.4785020	0.9941670
H	0.6834551	3.3228582	0.9104118
Pt	1.1657775	-2.4533583	-0.7014852
H	-0.3236836	-3.3644834	-0.4551471
H	1.6869267	-3.7409407	-1.4581241
H	2.8178787	-1.9683787	-0.9510306
H	2.1370526	2.5659080	-0.8664296
Pt	-1.5729108	-2.1448321	-0.2523640
H	-2.6040842	-3.3176134	-0.5568416
H	-1.5468535	-1.9234848	-2.0267675
H	-3.0423478	-1.1301716	-0.2610594
Pt	-0.2479293	-0.8324162	-2.3235960
H	2.9651443	0.4950542	-1.7846782
H	1.1103668	0.0959147	-3.0822866
H	-0.3788964	-1.3593395	-3.8140314
H	-1.3117967	3.1198103	-0.6908970
Pt	-1.2199877	1.6834966	-1.6088138

Pt	1.4916667	1.2590885	-1.8732867
H	0.0991159	2.3760996	-2.4179602
H	-1.3217737	0.4080824	-2.8777273

Isomer 2-7:(method: TPSS/def2-TZVP)

Energy = -1447.222106741

Pt	0.1490334	-0.1568781	0.2111583
Pt	-1.2328957	-1.3062183	2.2435786
H	-2.6831614	-0.4231415	1.8872050
H	-1.9074576	-2.0533032	3.4849534
H	1.5033496	-0.2395809	3.4536422
Pt	0.4950481	0.7778810	2.7889928
H	-0.7371316	-0.2154150	3.5321948
H	3.1923083	-1.2451461	1.2245128
Pt	2.8383219	-0.4690686	-0.1057159
H	3.3527015	0.9906789	0.7245864
Pt	1.8800793	2.0028320	0.7209838
H	1.8943148	1.8076908	2.4798464
Pt	-2.5760955	0.3100539	0.3189686
H	-2.8653933	1.2201201	-1.2378786
H	-2.5005924	1.8908037	1.1651128
H	-0.6848100	2.2464401	2.5376114
Pt	-0.8700589	2.3897601	0.8964240
H	0.6956683	3.2792780	0.8211298
Pt	1.0900329	-2.4997524	-0.7202750
H	-0.3873738	-3.4458948	-0.5870472
H	1.6199730	-3.8138163	-1.4394889

H	2.7672166	-2.0361596	-0.9186678
H	2.1714132	2.5241517	-0.9225310
Pt	-1.6369404	-2.2176151	-0.3759934
H	-1.8596225	-2.6735221	1.3376934
H	-1.6205132	-1.9894385	-2.1261406
H	-3.0951521	-1.2152398	-0.3697679
Pt	-0.2917184	-0.8909864	-2.3810444
H	2.9207642	0.3511917	-1.7977353
H	1.0680752	0.0163818	-3.1022590
H	-0.4040476	-1.4462632	-3.8692253
H	-1.3611091	3.0551234	-0.8014652
Pt	-1.2482377	1.6400776	-1.7237766
Pt	1.4792071	1.1994671	-1.9106985
H	0.1779539	2.3237047	-2.4394181
H	-1.3331502	0.3118034	-2.9994659

Isomer 2-8:(method: TPSS/def2-TZVP)

Energy = -1447.211860525

Pt	0.1778195	0.0316429	0.0922890
Pt	-1.2946901	-1.1172392	2.0718200
H	-2.0818704	-1.8120542	3.2686084
H	-1.9570198	-2.4240976	1.1047001
H	1.4124429	-0.1831459	3.2536555
Pt	0.4507925	0.9071206	2.6484736
H	-0.8405712	-0.0721498	3.3842978
H	3.3485179	-1.1672731	1.2645675
Pt	2.9660513	-0.4140593	-0.0591625

H	3.4541087	1.0549730	0.7509143
Pt	1.9900878	2.0676789	0.6790106
H	1.8894610	1.8794028	2.4523020
Pt	-2.4837977	0.6522578	0.0166037
H	-2.6509389	-0.0658789	1.5344716
H	-2.4393035	2.2600758	0.7179121
H	-0.7163733	2.3712690	2.3228896
Pt	-0.7236265	2.6348897	0.6552284
H	0.8576774	3.4098159	0.7330803
Pt	1.1682570	-2.3562732	-0.7013189
H	-0.3981073	-3.1910367	-0.6263894
H	1.7555691	-3.7919938	-1.0585609
H	2.8930215	-2.0208654	-0.7587469
H	2.3334128	2.5547189	-0.9578808
Pt	-1.6065853	-1.9290072	-0.5619656
H	-2.6326056	-3.0777279	-0.9536795
H	-1.5785487	-1.6638047	-2.3135156
H	-3.0503728	-0.8448292	-0.6333022
Pt	-0.1726003	-0.6458348	-2.5932780
H	3.1331257	0.5088864	-1.8288985
H	1.2791746	0.1661158	-3.2399814
H	0.8322966	-2.0322050	-2.3973017
H	-0.8910899	3.3363490	-0.9932488
Pt	-0.9399022	1.9266583	-2.0012145
Pt	1.6501466	1.2240346	-1.9187482
H	-4.0200285	1.0419208	-0.0636363
H	-1.1139310	0.7816657	-3.2899947

Isomer 2-9:(method: TPSS/def2-TZVP)

Energy = -1447.214862439

Pt	0.1689597	-0.2351301	0.3583965
Pt	-1.4730093	-1.1513128	2.2663667
H	-2.5759948	-1.5991583	3.3126733
H	-1.9825431	-2.5519305	1.3198056
H	1.2539402	-0.2942564	3.4653891
Pt	0.3378019	0.8321037	2.8345922
H	-1.0744785	-0.0191722	3.5280808
H	3.8656006	-1.2793088	0.8428596
Pt	3.0374528	-0.4922589	-0.2575448
H	3.3881002	0.9452949	0.6714719
Pt	1.8562888	1.9113871	0.7168456
H	1.8258239	1.6849554	2.5063380
Pt	-2.6066188	0.3720291	0.3411810
H	-2.9951905	1.1624067	-1.0937077
H	-2.5517981	2.0074526	1.1438264
H	-0.7569871	2.3657587	2.5628678
Pt	-0.8990037	2.4286659	0.9023730
H	0.6784858	3.2138191	0.8344834
Pt	1.2286797	-2.4574580	-0.8994071
H	-0.3082722	-3.2831453	-0.5316391
H	1.5005967	-1.1096579	0.7641488
H	2.9632504	-1.9938288	-1.1548633
H	2.1251681	2.4357644	-0.9244313
Pt	-1.6134449	-2.1379344	-0.3538916

H	-2.6162555	-3.3219324	-0.7203184
H	-1.6452966	-1.9333907	-2.1328543
H	-3.1064905	-1.1066384	-0.3579400
Pt	-0.3113549	-0.8796682	-2.4713454
H	2.9415424	0.4320602	-1.9020583
H	1.0325503	0.0543542	-3.2115266
H	0.7895823	-2.3815248	-2.5221139
H	-1.2859819	3.0365950	-0.7719069
Pt	-1.2430737	1.5727905	-1.6693301
Pt	1.4443905	1.1414005	-1.9216099
H	0.0324512	2.2762861	-2.4982144
H	-1.4248713	0.3545826	-2.9769965

Isomer 2-10:(method: TPSS/def2-TZVP)

Energy = -1447.227162993

Pt	0.1378493	-0.1560160	0.2475425
Pt	-1.2567001	-1.2813888	2.2478006
H	-2.8132057	-0.2456260	1.8982337
H	-0.1352331	-2.2367750	2.8044422
H	1.4747219	-0.0453729	3.5305137
Pt	0.4274609	0.9159556	2.8340554
H	-0.7838765	-0.1468410	3.5233468
H	3.0808409	-1.2480325	1.3275378
Pt	2.8003801	-0.4736616	-0.0198478
H	3.2336607	1.0056941	0.8195890
Pt	1.8108749	2.0583876	0.7353100
H	1.7794119	1.9556639	2.5454894

Pt	-2.6216387	0.3212020	0.3312279
H	-2.8809219	1.0592312	-1.2984809
H	-2.5943734	1.9321987	1.0798452
H	-0.8346021	2.2746903	2.5052075
Pt	-0.9511102	2.4353034	0.8469056
H	0.5870968	3.3183717	0.7764667
Pt	1.0736809	-2.5275353	-0.6919346
H	-0.4117007	-3.4502530	-0.5683168
H	1.6015477	-3.8128014	-1.4417198
H	2.7413600	-2.0354683	-0.8405985
H	2.0874383	2.4927892	-0.9251397
Pt	-1.6756071	-2.2438784	-0.3401351
H	-1.8337883	-2.6775277	1.3682167
H	-1.6831167	-2.0196058	-2.0782767
H	-3.1401518	-1.2230840	-0.2896170
Pt	-0.3158255	-0.9519091	-2.3598926
H	2.9019573	0.3351568	-1.7298891
H	1.0669735	-0.0637318	-3.0694005
H	-0.4208090	-1.5493033	-3.8202940
H	-1.3415769	3.0182654	-0.8271625
Pt	-1.2875877	1.5706793	-1.7647360
Pt	1.4448901	1.1383580	-1.8824949
H	0.1205525	2.2487696	-2.4769174
H	-1.3888724	0.3080948	-2.9968768

References

(S1) Wilson, A. J. C.; Prince, E. In *Internaltional tables of crystallography*; Doordrecht/Boston/London, Ed.; Kluwer Academic Publishers, 1999.

(S2) *CRC Handbook of Chemistry and Physics*, 66th ed.; CRC Press: Boca Raton, FL, 1986.

(S3) Schooss, D.; Blom, M. N.; Parks, J. H.; Issendorff, B. v.; Haberland, H.; Kappes, M. M. The Structures of Ag_{55}^{+} and Ag_{55}^{-}: Trapped Ion Electron Diffraction and Density Functional Theory. *Nano Lett.* **2005**, *5*, 1972–1977.

(S4) TURBOMOLE V7.7 2022, a development of University of Karlsruhe and Forschungszentrum Karlsruhe GmbH, 1989-2007, TURBOMOLE GmbH, since 2007; available from `https://www.turbomole.org`.

(S5) Balasubramani, S. G.; Chen, G. P.; Coriani, S.; Diedenhofen, M.; Frank, M. S.; Franzke, Y. J.; Furche, F.; Grotjahn, R.; Harding, M. E.; Hättig, C.; Hellweg, A.; Helmich-Paris, B.; Holzer, C.; Huniar, U.; Kaupp, M.; Marefat Khah, A.; Karbalaei Khani, S.; Müller, T.; Mack, F.; Nguyen, B. D.; Parker, S. M.; Perlt, E.; Rappoport, D.; Reiter, K.; Roy, S.; Rückert, M.; Schmitz, G.; Sierka, M.; Tapavicza, E.; Tew, D. P.; van Wüllen, C.; Voora, V. K.; Weigend, F.; Wodyński, A.; Yu, J. M. TURBOMOLE: Modular program suite for ab initio quantum-chemical and condensed-matter simulations. *J. Chem. Phys.* **2020**, *152*.

(S6) Eichkorn, K.; Treutler, O.; Öhm, H.; Häser, M.; Ahlrichs, R. Auxiliary Basis Sets to Approximate Coulomb Potentials. *Chem. Phys. Lett* **1995**, *240*, 283–290.

(S7) Eichkorn, K.; Weigend, F.; Treutler, O.; Ahlrichs, R. Auxiliary Basis Sets for Main Row Atoms and Transition Metals and Their Use to Approximate Coulomb Potentials. *Theor. Chem. Acc.* **1997**, *97*, 119–124.

(S8) Becke, A. D. Density-functional exchange-energy approximation with correct asymptotic behavior. *Phys. Rev. A* **1988**, *38*, 3098–3100.

(S9) Perdew, J. P. Density-functional approximation for the correlation energy of the inhomogeneous electron gas. *Phys. Rev. B* **1986**, *33*, 8822–8824.

(S10) Tao, J.; Perdew, J. P.; Staroverov, V. N.; Scuseria, G. E. Climbing the Density Functional Ladder: Nonempirical Meta–Generalized Gradient Approximation Designed for Molecules and Solids. *Phys. Rev. Lett.* **2003**, *91*, 146401.

(S11) Weigend, F.; Ahlrichs, R. Balanced Basis Sets of Split Valence, Triple Zeta Valence and Quadruple Zeta Valence Quality for H to Rn: Design and Assessment of Accuracy. *Phys. Chem. Chem. Phys.* **2005**, *7*, 3297–3305.

(S12) Andrae, D.; Häußermann, U.; Dolg, M.; Stoll, H.; Preuß, H. Energy-Adjusted ab initio Pseudopotentials for the Second and Third Row Transition Elements. *Theor. Chim. Acta* **1990**, *77*, 123–141.

(S13) Johansson, M. P.; Lechtken, A.; Schooss, D.; Kappes, M. M.; Furche, F. *Phys. Rev. A* **2008**, *77*, 053202–7.

(S14) Kelting, R.; Baldes, A.; Schwarz, U.; Rapps, T.; Schooss, D.; Weis, P.; Neiss, C.; Weigend, F.; Kappes, M. M. Structures of Small Bismuth Cluster Cations. *J. Chem. Phys.* **2012**, *136*, 154309–154319.

(S15) Bumüller, D.; Yohannes, A. G.; Kohaut, S.; Kondov, I.; Kappes, M. M.; Fink, K.; Schooss, D. Structures of Small Platinum Cluster Anions Pt_n^-: Experiment and Theory. *J. Phys. Chem. A* **2022**, *126*, 3502–3510.

(S16) Armbruster, M. K.; Weigend, F.; van Wüllen, C.; Klopper, W. Self-consistent treatment of spin–orbit interactions with efficient Hartree–Fock and density functional methods. *Phys. Chem. Chem. Phys.* **2008**, *10*, 1748–1756.

(S17) Caldeweyher, E.; Ehlert, S.; Hansen, A.; Neugebauer, H.; Spicher, S.; Bannwarth, C.; Grimme, S. A generally applicable atomic-charge dependent London dispersion correction. *J. Chem. Phys.* **2019**, *150*, 154122.

(S18) Jonsson, G. H. H. Improved tangent estimate in the nudged elastic band method for finding minimum energy paths and saddle points. *J. Chem. Phys.* **2000**, *113*, 9978–9985.

(S19) Reed, A. E.; Weinstock, R. B.; Weinhold, F. Natural population analysis. *J. Chem. Phys.* **1985**, *83*, 735–746.

(S20) Wiberg, K. B. Application of the pople-santry-segal CNDO method to the cyclopropylcarbinyl and cyclobutyl cation and to bicyclobutane. *Tetrahedron* **1968**, *24*, 1083–1096.

(S21) Schleyer, P. v. R.; Maerker, C.; Dransfeld, A.; Jiao, H.; van Eikema Hommes, N. J. R. Nucleus-Independent Chemical Shifts: A Simple and Efficient Aromaticity Probe. *J. Am. Chem. Soc* **1996**, *118*, 6317–6318.
